# Various Items

**Published:** 1880-02

**Authors:** 


GRAHAM BREAD.—Take the unbolted flour of wheat, wet it with lukewarm water, add salt and yeast, knead in enough more of this flour to make it stiff, add a little molasses, and, when risen, bake in medium-sized loaves.

ERYSIPELAS is said to be cured by applying to the part affected, a paste made of raw cranberries beaten.

KETTLES are cleansed of onion and other odors, by dissolving a teaspoonful of pearlash or saleratus in water, and washing them.

HAIR, removed by fevers and other sickness, is made to grow by washing the scalp with a strong decoction of sage leaves once or twice a day.

STINGS and bites are often instantaneously cured by washing them in hartshorn or turpentine.

FLIES DESTROYED.—A pint of sweet milk, a quarter of a pound of sugar, two ounces of ground pepper, simmer together for ten minutes, and place it about in shallow dishes. If this is true, there is no necessity for using poisonous articles about a house.

BOILING POTATOES.—It is said that in Ireland they always nick off a piece of the skin, put them in a pot of cold water, which is gradually heated, but never allowed to boil; cold water should be added as soon as the water begins to boil; when done, pour all the water off, cover the vessel with a cloth, and in a few minutes they are cool enough for use.

BUY the best articles for family use; for, although they cost more, good articles spend best.

SUGAR from Havana is always dirty, that from Brazil is clean, as also from Porto Rico and Santa Cruz. Refined sugars, whether loaf, crushed, or granulated, are the cheapest in the end.

LARD from a hog not over a year old, is the best, and should be hard and white.

BUTTER made in September and October is the best for winter use.

RICH cheese feels softer under the pressure of the finger. That which is very strong is neither very good nor healthy. To keep one that is cut, tie it up in a bag that will not admit flies, and hang it in a dry cool place. If mold appears on it, wipe it off with a dry cloth.

ANTS are kept out of drawers and other places by spirits of camphor.

BUTTER KEPT FRESH.—Take it as it comes from the churn, and wash the butter-milk thoroughly out of it, then dry the surface of the butter with a clean cloth, break into small pieces, and pack it solid into a crock. The air must be entirely expelled. Set the crock in a kettle half-filled with water, then place the kettle over the fire until the water boils. While boiling remove from the fire, and let the crock remain in the water until cold.

A MEDICINE.—Abernethy's prescription to a wealthy patient was: "Let your servant bring you three or four pails of water, and put it into a wash-tub; take off your clothes, get into it, and from head to foot rub yourself well with it, and you'll recover."

"This advice of yours seems very much like telling me to wash myself," said the patient.

"Well," said Abernethy, "it is open to that objection."

A PRESCRIPTION.—If you wake up thirsty, diet, that is, eat nothing; if you have diarrhea, be quiet, that is, do nothing, drink nothing; and if not better in twelve hours, send for a physician.

LIGHTNING-RODS, in cities, says Prof. Henry, of the Smithsonian Institute, should be connected with the water or gas-pipes underground, outside the building.

A BIT of glue dissolved in skim-milk and water, will restore old crape. Half a cranberry bound on a corn will soon kill it.

RED ANTS.—Wash your shelves down clean, and while damp rub fine salt on them quite thick, and let it remain on for a time, and they will disappear.

EYES.—If you must sew on black cloth at night, pin a piece of soft white paper along the seam, and sew through it; afterwards tear the paper away.

CAMOMILE.—The decoction of its leaves is said to destroy various insects; the living plant imparts health to other plants, often reviving drooping ones.

POTATOES contain nearly all their nutriment (the starch) very near the surface; the heart has but little; hence, let the peeling be the thinnest possible.

